# Interactions between Bacteriophages and Eukaryotic Cells

**DOI:** 10.1155/2020/3589316

**Published:** 2020-06-09

**Authors:** Ramendra Dirgantara Putra, Diana Lyrawati

**Affiliations:** Department of Pharmacy, Faculty of Medicine, Brawijaya University, Malang 65145, Indonesia

## Abstract

As the name implies, bacteriophage is a bacterium-specific virus. It infects and kills the bacterial host. Bacteriophages have gained attention as alternative antimicrobial entities in the science community in the western world since the alarming rise of antibiotic resistance among microbes. Although generally considered as prokaryote-specific viruses, recent studies indicate that bacteriophages can interact with eukaryotic organisms, including humans. In the current review, these interactions are divided into two categories, i.e., indirect and direct interactions, with the involvement of bacteriophages, bacteria, and eukaryotes. We discuss bacteriophage-related diseases, transcytosis of bacteriophages, bacteriophage interactions with cancer cells, collaboration of bacteriophages and eukaryotes against bacterial infections, and horizontal gene transfer between bacteriophages and eukaryotes. Such interactions are crucial for understanding and developing bacteriophages as the therapeutic agents and pharmaceutical delivery systems. With the advancement and combination of in silico, *in vitro*, and *in vivo* approaches and clinical trials, bacteriophages definitely serve as useful repertoire for biologic target-based drug development to manage many complex diseases in the future.

## 1. Introduction

Bacteriophages (phage, for short) were independently discovered as bacteria-specific viruses by the British microbiologist Frederick Twort in 1915 and French-Canadian scientist Felix d'Herelle in 1917 [1]. In the following years, bacteriophages were regarded as promising candidates for antimicrobial therapy, until the idea was abandoned in the western world because of the introduction of antibiotics [2]. Antibiotics were chosen over bacteriophages because they are fixed chemical compounds and are easy to manufacture. Nevertheless, bacteriophage research has been continued in some regions of the world, e.g., Georgia, Poland, and the Soviet Union, during the Second World War until today [3, 4]. With the emerging cases of antibiotic-resistant superbugs around the world, bacteriophages have regained scientific attention and are currently extensively studied as an alternative antimicrobial therapy [5]. Bacteriophage research has greatly expanded and encompasses many aspects of bacteriophage biology, from *in vitro*-level experiments to clinical trials.

The most recent clinical trial regarding the utilization of bacteriophage therapy in burn wounds infected by *Pseudomonas aeruginosa* had been conducted in the years 2015–2017. Despite the insufficient efficacy of the therapy compared with the control antibiotic sulfadiazine, because of a low amount of administered bacteriophages, the results sparked interest in the science community [6]. In February 2019, the US Food and Drug Administration has approved a clinical trial of bacteriophage therapy, initiated by the University of California, San Diego, School of Medicine and AmpliPhi Biosciences Corporation, in patients with ventricular assist devices infected by *Staphylococcus aureus* [7]. The bacteriophage therapy is administered via an intravenous route and is the first clinical trial of bacteriophage treatment in North America [7]. These two clinical trials and a few other unmentioned here mark new stages of the development of bacteriophages from the experimental stage to a regular commercially available therapy to eradicate highly resistant superbugs.

Other than the typical interactions with bacteria and its application in infectious disease, bacteriophages can also interact and affect eukaryotes in many unexpected ways [8]. In the current review, these interactions are divided into two categories: indirect and direct interactions. During the former, a bacteriophage affects a eukaryote by affecting eukaryotic-related bacteria, whereas, during the latter, the bacteriophage and eukaryote are in immediate contact. We present these interactions below and emphasize their impact on the future application of bacteriophages in the clinical setting.

## 2. Indirect Interactions between Bacteriophages and Eukaryotes

### 2.1. Bacteriophages Assist to Kill Bacterial Pathogens of Eukaryotes

The ability of bacteriophages to kill pathogenic bacteria of eukaryotes underpins the revived interest in bacteriophages as an alternative antimicrobial therapy. The detailed mechanisms used by bacteriophages to assist eukaryotes vary.

Barr et al. showed that bacteriophages, particularly T4 bacteriophages, act as non-host-derived immunity against bacterial invaders at human mucosal surfaces [9]. T4 phage binds weakly to a mucin glycoprotein, one of the essential building blocks of the mucus (secreted by the epithelial mucosa), using an immunoglobulin-like domain of Hoc protein. The weak binding provides additional protection to the epithelial cells by facilitating the T4 phage killing of and preventing colonization by bacterial pathogens [9]. The weak binding also maximizes the ability of T4 phage to kill bacteria by enabling it to move across mucosal surfaces in a subdiffusive fashion. Subdiffusive motion increases the probability of bacteriophage-bacterium encounters, as it allows the phage a thorough exploration of certain regions of the mucus [10].

Kaur et al. point out the ability of some bacteriophages to kill intracellular pathogens [11]. Intracellular pathogens are more dangerous and harder to eradicate than extracellular pathogens because they can evade the immune system by hiding inside the host cell. An early study showed that a broad-host-range lytic bacteriophage MR-5 is a promising candidate for phage therapy [11]. The bacteriophage kills methicillin-resistant *S. aureus* (MRSA) that had been phagocytosed by murine BALB/c macrophages, reducing the bacterial numbers so that the bacteria are handled more easily by macrophages. Furthermore, it also reduces the cytotoxic effect of *S. aureus* against macrophages by killing the bacteria. Therefore, MR-5 phage indirectly assists the macrophages in eliminating MRSA, providing major assistance, especially in the case of high bacterial loads. Instead of directly entering the macrophages, MR-5 phage uses *S. aureus* as a ride.

In a similar study, Zhang et al. found that another broad-host-range lytic bacteriophage, vB_SauM_JS25, can kill MRSA inside bovine epithelial cells (MAC-T bovine mammary epithelial cells) [12]. The authors reported that vB_SauM_JS25 phage can penetrate into the nucleus of MAC-T cell, although the underlying mechanism is not known. Subsequent investigations confirmed that several bacteriophages are indeed able to penetrate the eukaryotic cells, but not quite reach the nuclei [13, 14]. Bacteriophage penetration of the eukaryotic cells is discussed in detail as follows.

The occurrence of interactions between phages, bacteria, and eukaryotic cells raises the question about the effectiveness of phage therapy. A further study investigated the safety issues surrounding the effect of experimental bacteriophage therapy *in vitro* on the intracellular killing ability of granulocytes and monocytes [15]. The authors found that therapy involving T2, T4, and A3 bacteriophages does not affect the intracellular killing capability of the mentioned cells. A later investigation in humans [16] reached similar conclusions. Namely, that bacteriophage therapy, administered as a cocktail of several lytic bacteriophages, does not affect the killing ability of polymorphonuclear neutrophils and peripheral blood mononuclear cells during chronic infection caused by pathogenic *Escherichia coli*, *Enterococcus faecalis*, *P. aeruginosa*, and *S. aureus*, regardless of the route of administration and infection type. Furthermore, the bacteriophage therapy improved the killing ability of peripheral blood mononuclear cells during nonpathogenic *E. coli* B infections in patients who responded positively to the therapy. Based on these observations, the authors suggested that bacteriophage therapy is sufficiently safe to be employed in humans, especially in cases of chronic infection.

The studies confirm that in some systems bacteriophages can support the antibacterial activity of eukaryotic cells. Bacteriophages, however, may also interact indirectly with bacteria to harm eukaryotes, either by (1) disrupting a mutualistic relationship between eukaryotes and bacteria or by (2) supporting eukaryotic bacterial pathogen, as discussed in detail hereinafter.

### 2.2. Bacteriophages Disrupt the Mutualistic Microbial Equilibrium within the Human Body

The mutualistic relationship between microbes and humans is considered to be vital for maintaining physiological functions and homeostasis in the human body. The human host provides nutrition and habitat necessary to support bacterial growth. The microbiota produce metabolites which serve as signaling molecules to the gut, brain, immune and hormone system, and other functions of the host [17]. The relationship is important as the human body is a sanctuary of nearly 100 trillion microbes representing a wide range of species, especially within the digestive tract. The presence of bacteriophages that can kill bacteria, including beneficial bacteria, may shift the balance towards dysbiosis (maladaptation of the microbial composition), thus triggering diseases [18–21]. In other words, bacteriophages can also become human pathogens [22].

According to this hypothesis, bacteriophages may be the possible initiators of Parkinson's disease [23]. Parkinson's disease is marked by the accumulation of misfolded *α*-synuclein in dopaminergic neurons of the substantia nigra because of a depletion of the neurotransmitter dopamine. According to the proposed pathophysiologic pathway of Parkinson's disease, the misfolding of *α*-synuclein begins in the enteric nervous system and spreads gradually to the substantia nigra. The misfolding is thought to be the result of the absence of certain lactic acid bacteria, *Lactococcus* spp., in the gut, which are responsible for maintaining the proper levels of dopamine in the enteric nervous system [24]. L-DOPA, as part of Parkinson's disease drug regiment, affects the microbial population in the gut [25]. The analysis of the fecal samples from L-DOPA-naive Parkinson's disease patients showed alteration of microbiota [26] and phageota [23]. The gut population of *Lactococcus* spp. is 10 times lower than that in control individuals. The depletion of *Lactococcus* spp. is associated with the overabundance of specific lytic bacteriophages, *Lactococcus* bacteriophage 936 and *Lactococcus* virus c2. *Lactococcus* bacteriophages are thought to kill *Lactococcus* spp., thereby promoting the development of Parkinson's disease [23]. Since *Lactococcus* bacteriophages are frequently found in milk, cheese, and yogurt, the consumption of dairy products may lead to their high abundance in the human gut [27]. Additional data are required to validate such assumptions, perhaps by determining the average concentration of *Lactococcus* bacteriophage 936 and *Lactococcus* virus c2 in dairy products and investigating the correlation between consumption of dairy products containing these bacteriophages and the incidence of Parkinson's disease symptoms.

### 2.3. Bacteriophages Can Assist Bacterial Pathogens of Eukaryotes

Bacteriophages may assist in pathogenic bacterial infection in several different ways. Addy et al. investigated the involvement of filamentous phage *φ*RSS1 during *Ralstonia solanacearum* infection of tomato plants [28]. First, *φ*RSS1 enhances the *R. solanacearum* virulence via the attachment and accumulation of phage particles on the surface of the bacterial cell membrane. This subsequently increases the density of bacterial colonies and induces early expression of the gene for transcriptional regulator PhcA, responsible for the activation of many other virulence factors. These included the production of copious amounts of extracellular polysaccharide, which plays a vital role in expanding stem colonization in tomato. Moreover, *φ*RSS1 enhances the expression of PilA protein (type IV pilus), which further increases the twitch motility and attachment of bacterial colonies to plant cells. *R. solanacearum* strain infected by *φ*RSS1 causes complete wilt more rapidly in tomato plant (5 d after inoculation) than an uninfected strain (8 d after inoculation). Interestingly, this phenomenon could be more common in nature as similar observations were made for filamentous phage Xf2 which enhances the virulence of *Xanthomonas campestris* pv. *oryzae* [29].

Bacteriophages may also assist bacterial pathogens in biofilm formation [30]. Biofilm is an aggregation of bacteria in well-organized polymers. The extracellular matrix protects bacteria and allows them to attach to multiple surfaces within the host or nonliving objects. It is one of the key features of bacterial pathogens, enabling their survival in a harsh environment, evasion of the host's immune system, and promoting chronic infections [31, 32]. Accordingly, the existence of prophage SV1 in the *Streptococcus* (*St.*) *pneumoniae* genome is correlated with the bacterial ability to form biofilm [33]. SV1 prophage can spontaneously alter its life mode from static lysogenic to active lytic. Such alteration is sufficient to lyse small numbers of *St. pneumoniae* cells in a colony, providing extracellular DNA which serves as adhesion required for biofilm formation [34]. In other words, some *St. pneumoniae* cells are sacrificed via SV1-mediated lysis for biofilm construction to protect other cells. Although *St. pneumoniae* is already equipped with an autolytic mechanism, in the form of the lytic enzyme LytA [35], the presence of another lytic pathway (SV1-mediated) increases its capacity to form a biofilm. *St. pneumoniae* strain with SV1 prophage constructs thicker and denser biofilms in a shorter amount of time than *St. pneumoniae* without SV1 prophage [33].

Bacteriophages could also assist biofilm formation of other bacterial species like *P. aeruginosa* and *E. coli*. In cystic fibrosis, filamentous bacteriophages Pf and Fd assist *P. aeruginosa* and *E. coli* in constructing highly ordered biofilms containing stable liquid crystal structures [36]. The filamentous bacteriophages are continuously extruded from, but not lyse and kill, the bacterial host. The shape and negative charge of these filamentous bacteriophages appear to correlate with their ability to associate with polymers inside the biofilms. The resulting biofilms protect the bacteria from aminoglycoside antibiotics and dehydration and promote tighter attachment to surfaces than that of biofilms formed in the absence of bacteriophages [37]. Moreover, the association is maintained by a depletion attractive force, which depends on the ionic strength, polymer size, and polymer concentration. In a murine model of pneumonia, biofilms formed by bacteriophage Pf-aided *P. aeruginosa* allow the bacterium to remain in the lung by evading phagocytosis by macrophages and inhibiting inflammatory responses [38]. This interaction is quite remarkable because *P. aeruginosa* and *E. coli* act as hosts for bacteriophage Pf and Fd, respectively. Many laboratory and clinical strains of *P. aeruginosa* and *E. coli* harbor prophages Pf and Fd, which are activated when the bacteria form biofilms [38].

In studies of Bille et al., it was reported that prophage of a filamentous bacteriophage MDA*φ* (Meningococcal Disease-Associated) enhances the physical contact between *Neisseria meningitidis* cells during the attachment and colonization of epithelial cells in the human nasopharynx, before infection and penetration of the blood-brain barrier [39]. The initial attachment and colonization stage is mediated by a type IV pilus, creating the first layer of *N. meningitidis* cells that bind tightly to the apical surface of epithelial cells. Soon after the attachment, the cells multiply and create another layer of bacteria to form colonies. However, type IV pilus does not mediate the creation of this next layer because the expression of the pilus is repressed after the first layer is formed [40]. The authors found that *N. meningitidis* expresses and utilizes the MDA*φ* prophage as a replacement of type IV pilus. In other words, the bacteriophage particles are used like the pilus to create the next layer of bacteria attached to the first layer. Interestingly, the bacteriophage particles are secreted and directly embedded in the outer membrane of the bacteria without lysing or killing them. Many bacteriophage particles form huge bundles that aggregate the bacteria and protect the colonies from shear stress and the flow movement of epithelial cell cilia. The deletion of prophage MDA*φ* from the *N. meningitidis* genome results in aggregation and colonization failure [39].

The integral involvement of filamentous bacteriophages in promoting bacterial infection against eukaryotes reveals mutualistic behaviors of bacteria and bacteriophages. Consequently, these types of bacteriophages are becoming new potential targets for drug development against pathogenic bacteria. As a vital link between filamentous bacteriophages and resistant microbes is identified, perhaps there is a chance to combat these superbugs, not by directly attacking them but by destroying their allies.

## 3. Direct Interactions between Bacteriophages and Eukaryotes

Viruses (including bacteriophages) are obligate intracellular pathogens and are generally classified based on the respective host domain (bacteria, archaea, or eukaryotes) [41]. This classification is based on the general assumption that no virus can infect and interact directly with representatives of more than a single domain of life [42]. However, even though bacteriophages cannot infect domains of life other than bacteria, they can nonetheless interact directly and affect representatives of such other domains, especially eukaryotes. We describe some findings on direct interaction between bacteriophages and eukaryotes below.

### 3.1. Bacteriophages Can Penetrate and Disperse within a Eukaryotic Host

One important factor that enables bacteriophages to interact directly and affect eukaryotes is the ability to penetrate the cell membrane and spread freely within a eukaryotic host [43–45]. Studies involving monolayer epithelial cells from the gut (T84 and CaCo2), lung (A549), liver (Huh7), brain (hBMec), and kidney (MDCK) derived from human demonstrated that the bacteriophage penetration of cell relies on transcytosis, which involves the endomembrane systems of the eukaryotic cells, particularly the Golgi apparatus [14]. Transcytosis starts when a bacteriophage particle is engulfed by the cell membrane and transferred to the cytoplasm inside a small vesicle. The vesicle then transits inside the Golgi apparatus before being discharged on the other side of the same cell. The process repeated by the neighboring cells, thus enabling the bacteriophage particle crossing cell layers. This phenomenon has been observed for several bacteriophages, such as T4, T5, T7, SP01, SPP1, and P22 phages [14]. Detailed observations revealed that transcytosis of bacteriophages mainly proceeds in the apical to basolateral direction, with an estimated rate of 0.325 × 10^−12^ mL/(*μ*m^2^ h), and is considered a dose-dependent process [14].

Similar observations were made in another study using *E. coli* bacteriophage PK1A2 and human neuroblastoma cells kSK-N-SH that express a copious amount of polysialic acid on the membrane surface [13]. PK1A2 bacteriophage can bind to polysialic acid and enters kSK-N-SH cells by endocytosis. The bacteriophages accumulate in the late endosome compartment close to the perinuclear region, residing therein until gradual degradation by the cell, approximately 48 h after endocytosis. The binding between PK1A2 and polysialic acid of kSK-N-SH cell is specific, probably because of the structural similarity with the polysialic acid lipopolysaccharide of *E. coli* K1, the main host of PK1A2 bacteriophage. The binding is needed for the initiation of endocytosis and is temperature-dependent [13].

Bacteriophage penetration of the eukaryotic cell could explain the observation that bacteriophages are found in many multicellular eukaryotes, even in isolated regions, e.g., the human brain, which had been long considered sterile [46]. This observation provides valuable information on the pharmacokinetic aspects of bacteriophage treatment, which is vital in the context of using bacteriophages as an alternative antimicrobial therapy [47]. It also emphasizes the possibility of using bacteriophages as vectors in drug and gene delivery systems, including therapies targeted towards the brain tissue, gastrointestinal tract, and lung via systemic or local delivery [48–51]. Furthermore, it also provides a reasonable explanation of the mechanism of direct horizontal gene transfer (HGT) between phages and eukaryotes, discussed in a later section.

### 3.2. Bacteriophages Can Bind to and Hamper Metastasis of Cancer Cells

Bacteriophage can interact with cancer cells, inhibiting metastasis by using specific protein-protein configuration involving GP24 of the bacteriophage and integrin *β*3, HSP90 receptor, or other proteins of the cancer cells. This unusual interaction was investigated in melanoma and lung cancer cells in a mouse model (B16 and LLC cells, respectively) and in humans (HS294T and A549 cells, respectively) [52, 53]. The authors suggested that *in vitro* and *in vivo* metastasis of both cancer types is inhibited by the binding of bacteriophages T4 and HAP1 (a substrain of T4 bacteriophage). The hypothesis is that this inhibition is mediated by the specific interactions between GP24 to integrin *β*3 (*α*II*β*3/*α*v*β*3) on cancer cells. GP24 is a capsid protein with a specific Lys-Gly-Asp motif (the KGD motif). It forms a pentamer on every corner of the T4 and HAP1 head. Integrin *β*3 is a surface protein that is involved in various aspects of cell biology, such as tissue integrity, cell migration, cell survival, and angiogenesis [54]. In cancer cells, integrin *β*3 is highly abundant and regarded as one of the possible factors promoting metastasis [55, 56]. The KGD motif in GP24 is thought to participate in the binding between GP24 and integrin *β*3. Both T4 and HAP1 bacteriophages can bind integrin *β*3 via GP24, but HAP1 phage binding is significantly stronger than that of T4 phage [53]. The exact mechanism that triggers the inhibition of cancer metastasis after binding of GP24 and integrin *β*3 is unknown. Probably, the binding downregulates integrin *β*3 expression or prevents integrin *β*3 interaction with other proteins involved in some pathways related to cancer metastasis.

Detailed investigation revealed that the stronger binding by HAP1 phage is associated with a missense mutation in *hoc* gene, which encodes Hoc protein [57]. Hoc protein is a highly immunogenic outer capsid protein of the head of T4 and HAP1 phages. It has a dumbbell-like shape, which protrudes approximately 6 nm away from the capsid surface, including GP24 [58, 59]. Instead of a full-length Hoc protein like T4 phage, HAP1 phage produces a truncated Hoc protein because of a change of C496 residue to T, which subsequently changes the Gln166 codon into a stop codon (UAA). Hoc truncation in HAP1 phage possibly increases the probability of binding between GP24 and integrin *β*3, as it does not protrude away from the head and interferes with a direct contact between these two proteins. This notion is supported by a similar binding capacity of HAP1 phage and T2 bacteriophage, which does not possess the Hoc protein but does possess GP24 and KGD motif, to integrin *β*3 in cancer cells [57].

Investigation of T4 and HAP1 phage behavior in mouse harboring B16 melanoma cells yielded another interesting observation. It revealed that despite having a much greater affinity for integrin *β*3 in the cancer cells, HAP1 phage is removed more rapidly by Kupffer cells in the liver by phagocytosis than T4 phage [53]. It was proposed that HAP1 is more prone to phagocytosis because the short version of Hoc protein does not conceal the head capsid proteins, which are thus easily detected by Kupffer cells. Wild-type Hoc protein has four domains, three of which are similar to eukaryotic immunoglobulin. Domain 1 is similar to Fc receptors, domain 2 to that of the third immunoglobulin-like domain of Perlecan, and domain 3 to the V-set family of immunoglobulin superfamily [60]. Therefore, the Hoc protein probably evolved as a form of adaptation of T4 bacteriophages to avoid immune system recognition, thereby allowing them to survive inside eukaryotes.

Another study demonstrated that bacteriophages T4 and M13 can suppress the expression of HSP90 gene in human prostate cancer cells (PC3), responsible for the promotion of mitosis, DNA repair, and prevention of apoptosis and autophagy [61]. The suppression is mediated by the interaction of T4 and M13 phages with HSP90 receptors and results in apoptosis and autophagy of cancer cells. Similar to GP24-integrin *β*3 binding, the exact mechanism that promotes the downregulation of the expression of the HSP90 gene after T4 and M13 phage binding to HSP90 receptors is unknown. However, these findings highlight the unusual interactions between bacteriophages and eukaryotes and also present a new approach to explore the possible use of bacteriophages as anticancer agents.

Although the knowledge regarding interactions between *β*3 integrin and HSP90 receptor with bacteriophages is scarce, we speculate that the two proteins probably allow the bacteriophage to interact with eukaryotes in general, not only in cancer cells. This speculation is based on the information that *β*3 integrin and HSP90 receptors are found in many species, even though they may be not as abundant as in cancer cells. In other words, *β*3 integrin and HSP90 receptors are used by bacteriophages to interact with eukaryotes and vice versa. Further, this interaction probably initiates transcytosis of bacteriophages to eukaryotic cells via receptor-mediated endocytosis, similar to polysialic acid-mediated endocytosis of PK1A2 bacteriophage, as discussed earlier. If that is indeed the case, then a direct interaction between bacteriophages and eukaryotes is more common than currently assumed. Furthermore, *β*3 integrin, HSP90 receptor, and polysialic acid most probably are not the only types of mediators that mediate such direct interactions.

### 3.3. Human Cells Assist Bacteriophages in Infecting the Bacterial Host

The interaction between bacteriophages and human cells may be crucial for bacteriophage infection of bacteria. phiCDHS1 bacteriophage kills the pathogenic bacterium *Clostridium difficile* more rapidly when both are placed in a culture of a human colon cancer line HT-29 cells [62]. In other words, HT-29 cells seemingly help to maximize the killing ability of phiCDHS1 bacteriophage to eradicate C. *difficile*. This phenomenon is associated with the attachment of phiCDHS1 phage and C. *difficile* to HT-29 cells, which places phiCDHS1 phage and C. *difficile* cells in close contact, providing more opportunities for the phage to infect the bacterium. The propensity for this attachment is high as approximately 70% of phiCDHS1 phages were found attached to HT-29 cells in the study [62]. Further, the attachment is specific, as replacing HT-29 cells with HeLa cells (human cervical cancer line) led to no attachment and no increment of the phiCDHS1 killing capability or that of other C*. difficile* bacteriophages (phiCDHM3 and phiCDHM6). Since the attachment would enhance the eradication of *C. difficile* in the human gut and increase the population of phiCDHS1 phages, it can be classified as a direct mutualistic relationship between bacteriophages and humans. Unfortunately, the mediator of the attachment and the underlying mechanism are not clearly understood.

The attachment of phiCDHS1 phage to HT-29 cells supports our earlier speculation that direct interactions between bacteriophages and eukaryotes are more common in nature than currently proposed. Although the mechanism of the attachment remains unknown, it is possible that it is mediated by molecules with properties similar to those of *β*3 integrin, HSP90 receptor, and polysialic acid. In silico studies employing homology/comparative modeling, molecular docking, quantitative structure-activity relationship (QSAR) methods, and conformational analysis of bacteriophage, bacteria, and human proteome may identify the candidate protein(s) involved in such interactions.

### 3.4. Bacteriophages Engage in Direct HGT with Eukaryotes

HGT is defined as a transfer of genes from one organism to another unrelated organism [63, 64]. The transfer can occur between organisms from the same species, different species, and even organisms representing different domains. HGT occurs primarily in bacteria, by three pathways: transduction, transformation, and conjugation. The knowledge regarding HGT within the eukaryote domains is limited, but many lines of evidence indicate that it might also take place frequently in plants, animals, and humans [65]. HGT is common between bacteriophage and the bacterial host and also between bacteria and eukaryotes, particularly among obligate intracellular bacteria and their respective eukaryote host. At first glance, there is no direct HGT between bacteriophages and eukaryotes. However, such interactions occur, as it was shown for bacteriophage WO carrying several arthropod genes [42, 66]. Bacteriophage WO naturally infects *Wolbachia*. *Wolbachia*, in turn, is an intracellular bacterium of arthropods. By infecting *Wolbachia*, bacteriophage WO can be in contact with arthropod cells, which would allow HGT between them. Genes that are harbored by bacteriophage WO are collectively called eukaryotic association module. The module includes such genes as ones encoding latrotoxin-C-terminal domain, eukaryotic furin cleavage, ankyrin C-terminus, ankyrin and tetratricopeptide, and NACHT (Neuronal Apoptosis inhibitory protein, MHC Class II transcription activator, incompatibility locus protein from *Podospora anserina* HET-E, telomerase-associated protein TP1). Bacteriophage WO harbors all these genes, presumably to adapt to and survive within arthropod bodies and to efficiently infect *Wolbachia*. The mechanism that allows HGT between bacteriophage WO and arthropods is not fully understood. It is assumed that HGT might involve three different pathways: (1) direct gene transfer when bacteriophage WO enters the arthropod cells, (2) transfer mediated by *Wolbachia*, and (3) transfer mediated by other types of viruses that also infect the arthropods.

By contrast, several eukaryotes carry genes that relate to specific bacteriophages. Nematode and woodland strawberry carry an orthologous gene called *VP1*, which encodes a major capsid protein of *φ*Chp4, a *Chlamydia* bacteriophage from the Microviridae family [67]. It is possible that a fragment of bacteriophage gene(s) managed to integrate into the eukaryotes genomes, either by nonhomologues recombination or inserted during DNA replication.

The existence of a nuclear localization signal was demonstrated within Terminal Protein (TP) of several bacteriophages, e.g., Φ29, Nf, PRD1, Bam35, and Cp-1 [68]. The nuclear localization signal is a specific sequence that allows an uptake and delivery of proteins into the eukaryotic nucleus; thus, these TP-DNA molecules are a useful tool to amplify and subsequently deliver genes efficiently into the eukaryotic nucleus [69]. Meanwhile, TP is a protein used by bacteriophages to prime the DNA for replication. In that process, TP is covalently bound to the 5′-end of the DNA product. Therefore, following TP-mediated DNA replication, TP-bound bacteriophage DNA product has a nuclear localization signal and can enter the nucleus. This is another possible mechanism enabling direct HGT between bacteriophages and eukaryotes and also enabling eukaryotes to obtain bacteriophage genes. However, for such HGT to occur, the bacteriophage has to penetrate eukaryotic cells before releasing the TP-bound DNA. Therefore, bacteriophage penetration via transcytosis plays a vital role in allowing direct HGT to occur. This finding supports a new theory about the bacteriophage role in shaping the diversity of eukaryotic genomes.

## 4. Conclusion

Since their discovery in the early 20th century, bacteriophage characteristics and roles have triggered many basic and applied researches. Over time, the studies regarding bacteriophages extended from phage-bacteria interactions to interactions with nonbacterial cells, including cells of eukaryotes. These interactions include i.a. binding of phages with specific receptors on eukaryotic cells, transcytosis, and horizontal gene transfer. Furthermore, the potential roles of bacterial viruses in neurodegenerative disease and cancer have also been explored. Considering the remarkable diversity of phages and eukaryotes, more studies are required to identify new mechanisms of interactions and also to explain which interaction modes are common and which are unique. Not only are bacteriophages are exceptional microbes but they may also be used as research tools and valuable repertoire for biologic target-based drug development and drug delivery systems. Thus, interactions of bacterial viruses with eukaryotic cells are current and relevant research topic.

## References

[1] Wittebole X., De Roock S., Opal S. M. (2014). A historical overview of bacteriophage therapy as an alternative to antibiotics for the treatment of bacterial pathogens. *Virulence*.

[2] Roach D. R., Debarbieux L. (2017). Phage therapy: awakening a sleeping giant. *Emerging Topics in Life Sciences*.

[3] El-Shibiny A., El-Sahhar S. (2017). Bacteriophages: the possible solution to treat infections caused by pathogenic bacteria. *Canadian Journal of Microbiology*.

[4] Summers W. C. (2012). The strange history of phage therapy. *Bacteriophage*.

[5] Furfaro L. L., Payne M. S., Chang B. J. (2018). Bacteriophage therapy: clinical trials and regulatory hurdles. *Frontiers in Cellular and Infection Microbiology*.

[6] Jault P., Leclerc T., Jennes S. (2019). Efficacy and tolerability of a cocktail of bacteriophages to treat burn wounds infected by *Pseudomonas aeruginosa* (PhagoBurn): a randomised, controlled, double-blind phase 1/2 trial. *The Lancet Infectious Diseases*.

[7] Voelker R. (2019). Eye-tracking test approved to help diagnose concussion. *JAMA*.

[8] Chatterjee A., Duerkop B. A. (2018). Beyond bacteria: bacteriophage-eukaryotic host interactions reveal emerging paradigms of health and disease. *Frontiers in Microbiology*.

[9] Barr J. J., Auro R., Furlan M. (2013). Bacteriophage adhering to mucus provide a non-host-derived immunity. *Proceedings of the National Academy of Sciences*.

[10] Barr J. J., Auro R., Sam-Soon N. (2015). Subdiffusive motion of bacteriophage in mucosal surfaces increases the frequency of bacterial encounters. *Proceedings of the National Academy of Sciences*.

[11] Kaur S., Harjai K., Chhibber S. (2014). Bacteriophage-aided intracellular killing of engulfed methicillin-resistant *Staphylococcus aureus* (MRSA) by murine macrophages. *Applied Microbiology and Biotechnology*.

[12] Zhang L., Sun L., Wei R. (2017). Intracellular *Staphylococcus aureus* control by virulent bacteriophages within MAC-T bovine mammary epithelial cells. *Antimicrobial Agents and Chemotherapy*.

[13] Lehti T. A., Pajunen M. I., Skog M. S., Finne J. (2017). Internalization of a polysialic acid-binding *Escherichia coli* bacteriophage into eukaryotic neuroblastoma cells. *Nature Communications*.

[14] Nguyen S., Baker K., Padman B. S. (2017). Bacteriophage transcytosis provides a mechanism to cross epithelial cell layers. *mBio*.

[15] Kurzepa-Skaradzinska A., Lusiak-Szelachowska M., Skaradzinski G. (2013). Influence of bacteriophage preparations on intracellular killing of bacteria by human phagocytesin vitro. *Viral Immunology*.

[16] Jończyk-Matysiak E., Łusiak-Szelachowska M., Kłak M. (2015). The effect of bacteriophage preparations on intracellular killing of bacteria by phagocytes. *Journal of Immunology Research*.

[17] Schroeder B. O., Bäckhed F. (2016). Signals from the gut microbiota to distant organs in physiology and disease. *Nature Medicine*.

[18] De Sordi L., Khanna V., Debarbieux L. (2017). The gut microbiota facilitates drifts in the genetic diversity and infectivity of bacterial viruses. *Cell Host & Microbe*.

[19] Carding S. R., Davis N., Hoyles L. (2017). Review article: the human intestinal virome in health and disease. *Alimentary Pharmacology & Therapeutics*.

[20] Aggarwala V., Liang G., Bushman F. D. (2017). Viral communities of the human gut: metagenomic analysis of composition and dynamics. *Mobile DNA*.

[21] Shkoporov A. N., Hill C. (2019). Bacteriophages of the human gut: the “known unknown” of the microbiome. *Cell Host & Microbe*.

[22] Tetz G., Tetz V. (2018). Bacteriophages as new human viral pathogens. *Microorganisms*.

[23] Tetz G., Brown S. M., Hao Y., Tetz V. (2018). Parkinson’s disease and bacteriophages as its overlooked contributors. *Scientific Reports*.

[24] Klingelhoefer L., Reichmann H. (2015). Pathogenesis of Parkinson disease-the gut-brain axis and environmental factors. *Nature Reviews Neurology*.

[25] Fasano A., Visanji N. P., Liu L. W. C., Lang A. E., Pfeiffer R. F. (2015). Gastrointestinal dysfunction in Parkinson’s disease. *The Lancet Neurology*.

[26] Bedarf J. R., Hildebrand F., Coelho L. P. (2017). Erratum to: functional implications of microbial and viral gut metagenome changes in early stage L-DOPA-naive Parkinson’s disease patients. *Genome Medicine*.

[27] Mahony J., Murphy J., van Sinderen D. (2012). Lactococcal 936-type phages and dairy fermentation problems: from detection to evolution and prevention. *Frontiers in Microbiology*.

[28] Addy H. S., Askora A., Kawasaki T., Fujie M., Yamada T. (2012). The filamentous phage *ϕ*RSS1 enhances virulence of phytopathogenic *Ralstonia solanacearum* on tomato. *Phytopathology*.

[29] Kamiunten H., Wakimoto S. (1983). Cleavage of replicative form DNAs of filamentous phages Xf and Xf2 by restriction endonucleases. *Japanese Journal of Phytopathology*.

[30] Sutherland I. W., Hughes K. A., Skillman L. C., Tait K. (2004). The interaction of phage and biofilms. *FEMS Microbiology Letters*.

[31] Chen L., Wen Y. M. (2011). The role of bacterial biofilm in persistent infections and control strategies. *International Journal of Oral Science*.

[32] DeBardeleben H. K., Lysenko E. S., Dalia A. B., Weiser J. N. (2014). Tolerance of a phage element by *Streptococcus pneumoniae* leads to a fitness defect during colonization. *Journal of Bacteriology*.

[33] Carrolo M., Frias M. J., Pinto F. R., Melo-Cristino J., Ramirez M. (2010). Prophage spontaneous activation promotes DNA release enhancing biofilm formation in *Streptococcus pneumoniae*. *PLoS One*.

[34] Vilain S., Pretorius J. M., Theron J., Brözel V. S. (2009). DNA as an adhesin: *Bacillus cereus* requires extracellular DNA to form biofilms. *Applied and Environmental Microbiology*.

[35] Frias M. J., Melo-Cristino J., Ramirez M. (2009). The autolysin LytA contributes to efficient bacteriophage progeny release in *Streptococcus pneumoniae*. *Journal of Bacteriology*.

[36] Secor P. R., Sweere J. M., Michaels L. A. (2015). Filamentous bacteriophage promote biofilm assembly and function. *Cell Host & Microbe*.

[37] Secor P. R., Jennings L., Michaels L. (2016). Biofilm assembly becomes crystal clear-filamentous bacteriophage organize the *Pseudomonas aeruginosa* biofilm matrix into a liquid crystal. *Microbial Cell*.

[38] Secor P. R., Michaels L. A., Smigiel K. S. (2017). Filamentous bacteriophage produced by *Pseudomonas aeruginosa* alters the inflammatory response and promotes noninvasive infection in vivo. *Infection and Immunity*.

[39] Bille E., Meyer J., Jamet A. (2017). A virulence-associated filamentous bacteriophage of *Neisseria meningitidis* increases host-cell colonisation. *PLoS Pathogens*.

[40] Deghmane A.-E., Giorgini D., Larribe M., Alonso J.-M., Taha M.-K. (2002). Down-regulation of pili and capsule of *Neisseria meningitidis* upon contact with epithelial cells is mediated by CrgA regulatory protein. *Molecular Microbiology*.

[41] Nasir A., Forterre P., Kim K. M., Caetano-Anollés G. (2014). The distribution and impact of viral lineages in domains of life. *Frontiers in Microbiology*.

[42] Bordenstein S. R., Bordenstein S. R. (2016). Eukaryotic association module in phage WO genomes from Wolbachia. *Nature Communications*.

[43] Dabrowska K., Switala-Jelen K., Opolski A., Weber-Dabrowska B., Gorski A. (2005). Bacteriophage penetration in vertebrates. *Journal of Applied Microbiology*.

[44] Van Belleghem J., Dąbrowska K., Vaneechoutte M., Barr J., Bollyky P. (2018). Interactions between bacteriophage, bacteria, and the mammalian immune system. *Viruses*.

[45] Xu J., Xiang Y. (2017). Membrane penetration by bacterial viruses. *Journal of Virology*.

[46] Barr J. J. (2017). A bacteriophages journey through the human body. *Immunological Reviews*.

[47] Malik D. J., Sokolov I. J., Vinner G. K. (2017). Formulation, stabilisation and encapsulation of bacteriophage for phage therapy. *Advances in Colloid and Interface Science*.

[48] Ksendzovsky A., Walbridge S., Saunders R. C., Asthagiri A. R., Heiss J. D., Lonser R. R. (2012). Convection-enhanced delivery of M13 bacteriophage to the brain. *Journal of Neurosurgery*.

[49] Namdee K., Khongkow M., Boonrungsiman S. (2018). Thermoresponsive bacteriophage nanocarrier as a gene delivery vector targeted to the gastrointestinal tract. *Molecular Therapy-Nucleic Acids*.

[50] Xu H., Bao X., Wang Y. (2018). Engineering T7 bacteriophage as a potential DNA vaccine targeting delivery vector. *Virology Journal*.

[51] Huh H., Wong S., Jean J. S., Slavcev R. (2019). Bacteriophage interactions with mammalian tissue: therapeutic applications. *Advanced Drug Delivery Reviews*.

[52] D[aogon]browska K., Opolski A., Wietrzyk J. (2005). Activity of bacteriophages in murine tumor models depends on the route of phage administration. *Oncology Research Featuring Preclinical and Clinical Cancer Therapeutics*.

[53] Dabrowska K., Opolski A., Wietrzyk J. (2004). Antitumor activity of bacteriophages in murine experimental cancer models caused possibly by inhibition of beta3 integrin signaling pathway. *Acta virologica*.

[54] Avraamides C. J., Garmy-Susini B., Varner J. A. (2008). Integrins in angiogenesis and lymphangiogenesis. *Nature Reviews Cancer*.

[55] Danhier F., Le Breton A., Préat V. (2012). RGD-based strategies to target alpha(v) beta(3) integrin in cancer therapy and diagnosis. *Molecular Pharmaceutics*.

[56] Pan B., Guo J., Liao Q., Zhao Y. (2018). *β*1 and *β*3 integrins in breast, prostate and pancreatic cancer: a novel implication. *Oncology Letters*.

[57] Dabrowska K., Zembala M., Boratynski J. (2007). Hoc protein regulates the biological effects of T4 phage in mammals. *Archives of Microbiology*.

[58] Fokine A., Chipman P. R., Leiman P. G., Mesyanzhinov V. V., Rao V. B., Rossmann M. G. (2004). Molecular architecture of the prolate head of bacteriophage T4. *Proceedings of the National Academy of Sciences*.

[59] Leiman P. G., Kanamaru S., Mesyanzhinov V. V., Arisaka F., Rossmann M. G. (2003). Structure and morphogenesis of bacteriophage T4. *Cellular and Molecular Life Sciences (CMLS)*.

[60] Fokine A., Islam M. Z., Zhang Z., Bowman V. D., Rao V. B., Rossmann M. G. (2011). Structure of the three N-terminal immunoglobulin domains of the highly immunogenic outer capsid protein from a T4-like bacteriophage. *Journal of Virology*.

[61] Sanmukh S. G., Dos Santos S. A. A., Felisbino S. L. (2017). Natural bacteriophages T4 and M13 down-regulates Hsp90 gene expression in human prostate cancer cells (PC-3) representing a potential nanoparticle against cancer. *Virology Research Journal*.

[62] Shan J., Ramachandran A., Thanki A. M., Vukusic F. B. I., Barylski J., Clokie M. R. J. (2018). Bacteriophages are more virulent to bacteria with human cells than they are in bacterial culture; insights from HT-29 cells. *Scientific Reports*.

[63] Jeong H., Arif B., Caetano-Anollés G., Mo Kim K., Nasir A. (2019). Horizontal gene transfer in human-associated microorganisms inferred by phylogenetic reconstruction and reconciliation. *Scientific Reports*.

[64] Schneider C. L., Harper D. (2017). Bacteriophage-mediated horizontal gene transfer: transduction. *Bacteriophages: Biology, Technology, Therapy*.

[65] Koskella B., Taylor T. B. (2018). Multifaceted impacts of bacteriophages in the plant microbiome. *Annual Review of Phytopathology*.

[66] Bordenstein S. R., Marshall M. L., Fry A. J., Kim U., Wernegreen J. J. (2006). The tripartite associations between bacteriophage, Wolbachia, and arthropods. *PLoS Pathogens*.

[67] Rosenwald A. G., Murray B., Toth T., Madupu R., Kyrillos A., Arora G. (2014). Evidence for horizontal gene transfer between *Chlamydophila pneumoniae* and Chlamydia phage. *Bacteriophage*.

[68] Redrejo-Rodríguez M., Salas M. (2014). Multiple roles of genome-attached bacteriophage terminal proteins. *Virology*.

[69] Redrejo-Rodriguez M., Muñoz-Espín D., Holguera I., Mencía M., Salas M. (2013). Nuclear localization signals in phage terminal proteins provide a novel gene delivery tool in mammalian cells. *Communicative & Integrative Biology*.

